# AI-Driven BCR Modeling for Precision Immunology

**DOI:** 10.3390/ijms27073296

**Published:** 2026-04-05

**Authors:** Tao Liu, Xusheng Zhao, Fan Yang

**Affiliations:** 1 Department of Biomedical Engineering, Southern University of Science and Technology, Shenzhen 518055, China; t.liu@siat.ac.cn; 2 State Key Laboratory of Quantitative Synthetic Biology, Shenzhen Institute of Synthetic Biology, Shenzhen Institutes of Advanced Technology, Chinese Academy of Sciences, Shenzhen 518055, China; xszhao@link.cuhk.edu.hk; 3 Faculty of Synthetic Biology, Shenzhen University of Advanced Technology, Shenzhen 518107, China

**Keywords:** antibody repertoire, BCR, deep learning, machine learning, immunogenetics

## Abstract

The B cell receptor (BCR) repertoire captures an individual’s immunological history and antigen-driven evolution within a vast, high-dimensional sequence space. Although bulk and single-cell adaptive immune receptor repertoire sequencing (AIRR-seq) now enables deep profiling of BCR diversity, interpreting these datasets remains challenging due to strong inter-individual heterogeneity, nonlinear sequence–structure–function relationships, dynamic clonal evolution, and the rarity of functionally relevant clones. Artificial intelligence (AI) provides a conceptual and computational framework for addressing these challenges. Here, we summarize how advanced deep learning architectures, including antibody-specific language models, graph neural networks (GNNs), and generative frameworks, uncover clonal topology, structural features, and antigen-binding semantics. We further highlight applications in cancer, infectious disease, and autoimmunity. Finally, we propose a closed-loop framework that integrates multimodal datasets, interpretable AI, and iterative experimental validation to advance predictive immunology and accelerate therapeutic antibody discovery.

## 1. Introduction

The adaptive immune system maintains a lifelong record of antigen exposure through its extraordinarily diverse BCR repertoire. High-throughput immune repertoire sequencing now enables profiling of this diversity at single-cell resolution and with paired-chain information, generating datasets that capture V(D)J usage, somatic hypermutation (SHM) patterns, clonal lineage structure, and antigen-driven selection. Yet, despite this rapid expansion in data volume and resolution, our ability to interpret BCR repertoires to reveal antigen specificity and functional potential has not kept pace.

A fundamental challenge lies in the intrinsic complexity of BCR repertoires. Receptors vary in Complementarity-determining region (CDR) length, mutation rate, V(D)J gene usage, and structural conformation, creating a nonlinear and highly individualized mapping between sequence and function. Protective or pathogenic clones are often rare and embedded within the long tail of the repertoire. Clonal evolution unfolds through branching, graph-like trajectories that cannot be adequately captured by traditional motif- or similarity-based analyses. These limitations have constrained most repertoire studies to descriptive statistics rather than predictive or mechanistic modeling.

The convergence of adaptive immune receptor repertoire sequencing (AIRR-seq), structural modeling, and artificial intelligence (AI)—particularly antibody-specific language models—is catalyzing a paradigm shift. Deep learning architectures can now decipher the latent grammar of immune sequences, reconstruct clonal evolution, infer antigen specificity, and de novo-generate antibodies with optimized properties. Thus, AI represents more than an analytical upgrade; it constitutes a conceptual reframing of immune receptors as a learnable and generative biological language.

Beyond immunology, machine learning and artificial intelligence are rapidly transforming a wide range of scientific disciplines [1,2]. In pharmaceutical research, AI has accelerated drug discovery and optimization by enabling virtual screening and molecular design [3]. In materials science and mineral exploration, data-driven models are used to predict novel compounds and optimize resource identification [4]. Similarly, in food science, AI is increasingly applied to protein design, fermentation optimization, and quality control [5]. These advances collectively illustrate a broader paradigm shift toward data-driven discovery, within which immune repertoire analysis represents a complex and rapidly evolving research area.

**Figure 1:**
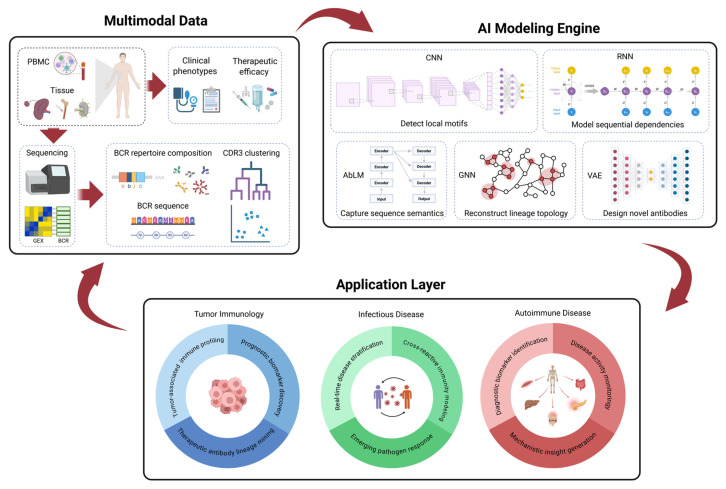
A closed-loop intelligent immune modeling and intervention system. Multimodal data layer: Next-generation sequencing of clinical samples (e.g., PBMCs, tissue) yields paired-heavy–light-chain BCR repertoires and transcriptomic data. These are integrated with associated clinical phenotypes to establish a standardized, interoperable data foundation. AI modeling engine: Diverse neural architectures are employed to decode repertoire complexity: convolutional neural networks (CNNs) detect local motifs and short-range affinity features; recurrent neural networks (RNNs) model sequential dependencies and SHM-driven mutation trajectories; antibody language models (AbLMs) capture global sequence semantics and long-range residue dependencies; graph neural networks (GNNs) reconstruct clonal lineage topology and selection dynamics; and generative frameworks (e.g., variational autoencoders (VAEs) or diffusion models) explore the BCR latent space to simulate affinity maturation and design novel antibodies or immunogens. Application layer: The framework addresses critical challenges across tumor immunology, infectious disease, and autoimmunity. Applications range from therapeutic lineage mining and biomarker discovery to mechanistic modeling of pathogen responses. Experimental or clinical outcomes are iteratively fed back into model refinement, establishing a closed loop from data acquisition to prediction and immune intervention. This figure was created using BioRender.com (Liu, T., 2026; https://BioRender.com/1j1b0k6 (accessed on 24 December 2025)).

In this review, we outline how AI models decode BCR sequence semantics, capture clonal topology, predict antigen specificity, and generate novel antibody candidates. We also critically examine key challenges, including data heterogeneity, model interpretability, rare-clone detection, and cross-cohort generalization. Finally, we propose a closed-loop framework integrating AI predictions with experimental validation. As illustrated by the system in Figure 1, this approach aims to advance predictive immunology and accelerate therapeutic discovery.

## 2. Opportunities and Challenges in BCR Repertoire Analysis

The B cell receptor (BCR) acts as the adaptive immune system’s primary sensor for recognizing threats. The diversity of the BCR repertoire dictates an individual’s capacity to recognize and respond to a vast array of antigens, serving as the foundation of immune defense. The mechanisms underlying BCR diversity, including V(D)J recombination, SHM, and antigen-driven selection in the germinal centers, have long been elucidated in classical immunology [6,7,8,9,10,11]. With the advent of high-throughput and single-cell immune repertoire sequencing, BCR repertoires can now be profiled at paired-chain and longitudinal resolution [12,13,14,15]. However, the exponential growth of high-resolution datasets is rapidly outpacing our interpretative capacity, pushing the field toward a critical inflection point.

BCR repertoires are information-rich archives of immune history that reflect responses to infection, vaccination, and tissue microenvironmental cues. They also harbor aberrant clones involved in autoimmune tolerance breakdown, tumor-driven selection, and long-lived vaccine-induced protection [16,17,18,19,20,21,22,23,24]. Extracting biologically meaningful and predictive patterns from this sea of sequences has thus become a central challenge linking fundamental immunology with precision medicine.

## 3. Dual Barriers in BCR Repertoire Analysis: Data Scale and Biological Complexity

While high-throughput sequencing has granted access to BCR diversity at an unprecedented resolution [12,13,25,26], transforming this vast data into actionable immunological insights is impeded by two fundamental bottlenecks, outlined below.

### 3.1. Data Dimensionality and Technical Variability

BCR repertoire datasets are characterized by extreme dimensionality, sparsity, and heterogeneity. A single sample can contain tens of thousands to millions of distinct receptor sequences, with substantial variation in length, mutation sites, and gene usage, creating a complex and nonlinear immune landscape [26,27,28]. This high-dimensional nature often leads to feature redundancy, unstable training processes, and model overfitting, particularly when the sample size is limited. Technical biases, ranging from platform-specific variations (e.g., UMI standardization and error correction) to fluctuations in sequencing depth, introduce significant batch effects, undermining model stability and cross-study comparability.

Moreover, the inherent limitation of data representation is that BCR function is highly dependent on its three-dimensional conformation, while the linear sequence information we obtain provides only an incomplete proxy for structure–function relationships. Consequently, traditional methods relying on sequence similarity often fail to capture critical nonlinear structure–function relationships [29,30,31,32].

### 3.2. Biological Individuality and Immune System Complexity

BCR repertoires are profoundly shaped by host-specific factors including genetic variants, antigen-exposure history, and age, resulting in highly individualized profiles [33,34]. Public clones shared across individuals are rare and unstable, complicating generalizable patterns. Furthermore, BCR evolution within germinal centers follows nonlinear, graph-like topologies involving branching and parallel mutation, which are poorly captured by tree- or similarity-based methods [35]. Functionally, rare but indispensable clones, such as those with high somatic hypermutation or polyreactivity, are often numerically eclipsed by dominant populations [28,36]. These clones are frequently missed by conventional frequency- or motif-driven analytical frameworks.

Together, these technical and biological barriers constrain the extraction of generalizable and predictive immunological patterns from repertoire data, underscoring the need for more expressive and biologically informed analytical frameworks [37].

## 4. Machine Learning and Deep Learning: A New Paradigm for BCR Analysis

Although high-throughput sequencing has enabled unprecedented depth in observing the BCR sequence space, the mapping between sequence, structure, and function remains highly nonlinear, rendering traditional analytical approaches inadequate for capturing the inherent complexity of the immune system. The introduction of artificial intelligence represents a profound paradigm shift in BCR repertoire analysis, moving beyond shallow analyses based on local motifs or sequence similarity toward a unified framework capable of modeling high-dimensional representations, graph-based topological relationships, and generative design. Crucially, the value of AI models lies not only in improved predictive performance, but in their capacity to learn the latent sequence semantics, structural grammar, and evolutionary logic that govern immune organization.

Early deep learning approaches, such as convolutional neural networks (CNNs), demonstrated the ability to detect local structural motifs within BCRs. CNNs can autonomously identify CDR1/2/3 motifs, short-range affinity determinants, and key contact residues, supporting receptor classification or SHM hotspot detection [38,39,40]. However, their limited capacity to model long-range dependencies constrains complex functional predictions.

While recurrent neural networks (RNNs), including LSTMs and GRUs, can model sequential dependencies and mutation trajectories to capture SHM-driven lineage preferences, they are inherently limited. Recurrent neural networks are known to exhibit inherent sequential processing characteristics and potential gradient instability, which may limit parallelization efficiency compared with attention-based architectures. Although several studies have successfully applied RNN-based models to large immune repertoire datasets [41,42], these architectural properties may pose scalability challenges as dataset sizes continue to expand. They are more suitable for sequence refinement than for full-spectrum prediction [43].

The true paradigm shift arises with antibody language models (AbLMs) based on the Transformer architecture. Trained on millions of receptor sequences using self-supervised learning, AbLMs capture residue substitution preferences, hidden structural rules, SHM accumulation patterns, and even epitope-level semantics. Unlike RNNs, their global attention mechanisms effectively resolve long-range residue co-dependencies, significantly enhancing antigen specificity prediction, affinity modeling, and functional annotation. By mapping discrete BCR sequences into continuous latent spaces, AbLMs facilitate cross-individual and even cross-species repertoire integration, effectively reframing the BCR repertoire as a learnable immune language [44,45].

Yet, sequence modeling alone fails capture the graph-like nature of clonal evolution. Germinal center responses involve complex networks of branching and convergence. Graph neural networks (GNNs) are uniquely suited to this task: by representing sequences as nodes and mutations as edges, GNNs model mutation interactions, lineage topology, and selection pressure. Compared to phylogenetic methods, GNNs more accurately reconstruct lineage trees and identify high-impact clonal variants, including rare but functionally critical ones [35,46,47].

Meanwhile, structure-aware models fill the “3D gap” between sequence and function. Leveraging antibody–antigen docking data, structural templates, or AlphaFold-like predictors, these models integrate sequence, conformation, and function. Advances in structure prediction, such as AlphaFold 3 [48], provide increasingly accurate structural priors for antibody–antigen complexes. While the integration of these structural representations with AbLM embeddings represents an emerging direction, systematic evaluations of their combined impact on downstream predictive performance remain limited.

Most futuristically, generative frameworks, including variational autoencoders (VAEs), diffusion models, and antibody-specific Transformers, enable deep exploration of the BCR latent space [49,50,51,52,53]. These models can simulate affinity maturation, design de novo antibodies, or propose theoretically valid but evolutionarily unobserved responses. They transition AI from a purely analytical tool into a robust platform for computational immunological experimentation. Challenges persist, particularly in integrating structural constraints and balancing multiple objective optimizations, such as maximizing affinity and minimizing immunogenicity, which are important for clinical translation.

Despite the broad applicability of these architectures, their performance and suitability vary significantly across different analytical scenarios. CNN-based models are computationally efficient and effective at detecting local sequence motifs but are inherently limited in capturing long-range dependencies [54]. RNNs partially address this limitation by modeling sequential dynamics; however, they often suffer from vanishing gradients and limited scalability for long immune sequences [55]. Transformer-based models overcome these constraints through self-attention mechanisms, enabling the modeling of global contextual relationships, but require substantially larger datasets and computational resources [45]. GNNs further extend modeling capacity by incorporating relational and topological information, making them particularly suitable for clonal lineage and network-based analyses, although their performance depends heavily on the quality of graph construction [47]. In contrast, generative models prioritize exploration of the sequence space and design capability, yet face challenges in ensuring biological validity and experimental feasibility. Collectively, these observations underscore that model selection should be task-specific and data-dependent, rather than relying on a single dominant architecture.

Together, CNNs (local motifs), RNNs (sequential dynamics), Transformers (sequence semantics), GNNs (clonal topology), structure-aware models (3D inference), and generative networks (design) form a multiscale hierarchy. A comprehensive summary of the application scenarios of these models is provided in Table 1. Through this integrated framework, the BCR repertoire transforms from a static sequence archive into a dynamic, learnable, and designable system, opening new frontiers in immunology.

## 5. Core Applications of AI in BCR Analysis

In the preceding section, we outlined how AI establishes a new analytical paradigm for BCR repertoire analysis, effectively addressing high dimensionality, pronounced heterogeneity, and complex nonlinear patterns. Building on this foundation, we now focus on a central task that transcends immunological contexts, namely antigen specificity prediction and immunogen design. We explore how AI is operationalized across three major disease domains: cancer, infectious disease, and autoimmunity. Despite distinct disease mechanisms and clinical settings, these applications share a unifying logic: extracting predictive, mechanistic, and design-relevant patterns from massive and noisy BCR datasets. AI is driving the field from static description toward dynamic prediction, mechanistic modeling, and even forward-looking immune engineering.

### 5.1. Antigen Specificity Prediction and Immunogen Design

Predicting antigen specificity and affinity from receptor sequences, and ultimately designing novel antibodies or immunogens, remains a pivotal challenge with profound translational implications. The underlying difficulty stems from the nonlinear, context-dependent mapping from sequence to structure to function, compounded by high inter-individual variability. Early antibody-specific language models such as AbLang demonstrated the feasibility of learning antibody-specific sequence embeddings, which have since been widely adopted for downstream fine-tuning tasks [56]. Transformer-based AbLMs, such as AntiBERTa and IgLM, have emerged as robust solutions [57,58]. Trained on millions of sequences in an unsupervised fashion, these models capture generalized sequence semantics that reflect conserved structural and functional patterns across antigen classes [59]. Fine-tuning with supervised data further enables the accurate prediction of antigen-binding potential, affinity shifts, and cross-reactivity, surpassing conventional feature-engineering methods in both generalization and interpretability. Recent explainable antibody language models further advance antigen specificity prediction by integrating curated datasets and interpretable attention mechanisms. For instance, Wang et al. developed a specificity-focused language model trained on curated influenza hemagglutinin antibodies, providing both predictive performance and residue-level interpretability [60].

Recent structure-guided Transformers, which integrate attention mechanisms with 3D spatial constraints, enhance the localization of critical functional residues, particularly within the CDRH3 region. These models analyze sequence-level patterns associated with antigen recognition and prioritize candidate variants with higher predicted binding likelihood. While such analyses do not perform explicit structural reverse inference or de novo immunogen design, they may provide indirect guidance for rational immunogen development by narrowing the search space of functionally relevant sequences [61]. Furthermore, hybrid approaches combining graph convolutional networks (GCNs) with structural predictors such as AlphaFold now allow simultaneous encoding of sequence embeddings and predicted conformations, enabling computational exploration of epitope-recognition patterns and potential immunogenicity profiles, although validation across diverse populations remains limited [62,63]. Such strategies are invaluable for high-variability antigens, such as HIV and SARS-CoV-2, potentially supporting rational vaccine design strategies and predictive immunodiagnostic research, pending further experimental validation.

Generative frameworks, including VAEs, diffusion models, and antibody-specific Transformers, extend this paradigm by enabling the exploration of the BCR latent space [49,50,51,52,53]. Conditioning latent representations on desired properties allows generation of candidate antibodies with targeted binding profiles, dramatically accelerating the optimization and screening pipeline. Some examples of generative models for antibody and immunogen design are summarized in Table 2. This transition transforms AI from a purely analytical tool into a platform for computational immunoengineering.

Despite their promise, generative models for antibody and immunogen design still face important translational limitations [52,64]. First, computationally generated sequences are not necessarily structurally valid, as sequence-level plausibility does not guarantee correct folding, stable conformations, or productive antigen engagement. Second, experimental validation remains a major bottleneck, because only a small fraction of in silico candidates can typically be synthesized and tested in vitro or in vivo. Third, therapeutic development requires not only binding activity but also rigorous developability assessment, including stability, solubility, aggregation resistance, polyreactivity/off-target binding, and expression yield, which are not consistently optimized in current generative frameworks. Finally, reliable prediction of immunogenicity remains challenging, particularly because immune responses are influenced by host-dependent and context-specific factors that are still insufficiently captured by available training datasets and predictive models. These limitations indicate that generative AI should currently be viewed as a powerful proposal engine that must be tightly integrated with empirical validation rather than a standalone solution for therapeutic design.

**Table 2:** Representative applications of generative models in antibody and immunogen design.

Model Type	Current Applications	Advantages	Future Directions/Potential Extensions	References
VAE	Immunoglobulin-fold backbone (scaffold) generation/structure-constrained design	High controllability; suitable for structure-constrained design	Incorporating structural information for end-to-end optimization; enhancing generalization for rare epitope design	[49]
Diffusion	Epitope and antigen design	High-fidelity generation; capable of modeling complex distributions	Integrating immune escape prediction and antigen variation simulation to improve clinical relevance of generated antigens	[51,53]
GPT-like models	Antibody language generation	Preserves semantic structure; high naturalness	Cross-species transfer learning of antibody repertoires; multimodal generation incorporating structural data	[58,65,66]

Abbreviations: VAE, variational autoencoder; GPT, generative pre-trained transformer.

In summary, AI has shifted antigen specificity analysis from descriptive cataloging to predictive modeling and rational antibody design, providing a powerful computational engine for broad-spectrum vaccines and personalized therapeutics.

### 5.2. Tumor Immunology: From Prognostic Markers to Predictive Response Models

The role of B cells in tumor immunity has historically been underestimated. Today, the integration of AI is catalyzing a reevaluation of humoral immunity in oncology, particularly focusing on three critical tasks: (1) immune profiling and clonal identification of tumor-associated BCRs within the tumor microenvironment (TME); (2) extraction of prognostic biomarkers linked to clinical outcomes and immunotherapy response; and (3) mining of functional antibody lineages for therapeutic development.

At the identification level, machine learning models have laid the groundwork for distinguishing tumor-specific immune signatures. Early studies demonstrated that supervised classifiers could distinguish BCR repertoire composition between tumor and adjacent normal tissue [67]. As modeling techniques have evolved, ensemble learning algorithms (e.g., XGBoost, random forests) and neural networks have been applied to identify disease-specific BCR combinations in malignancies like lymphomas, enabling immune subtyping of B cell populations in the TME and assisting diagnostic efforts [68].

At the prognostic level, BCR repertoire features are emerging as powerful biomarkers. The abundance and activation state of tumor-infiltrating B cells (TIL-Bs) have been associated with immune checkpoint therapy response and survival in solid tumors such as melanoma [69]. Moreover, dynamic repertoire features, such as BCR diversity indices and clonal lineage reconstruction, have shown promise as predictive markers for PD-1/PD-L1 immune checkpoint blockade outcomes [70]. Deep learning models can further refine this analysis by modeling sequence and structural determinants of antigen binding, thereby uncovering BCR features that are predictive of treatment response [71].

Perhaps the most translationally promising avenue is the discovery of shared tumor-reactive BCR lineages across individuals. A key phenomenon here is convergent selection, where unrelated patients independently evolve antibodies with similar antigen-binding properties under common tumor pressure. Natural language processing (NLP)-inspired models such as Immune2vec capture these convergent patterns by learning semantic similarity across patient repertoires, uncovering public structures with the potential to form the basis of next-generation tumor antibody libraries [44].

Ultimately, AI is reshaping tumor immunology from merely recognizing tumor-associated features to predicting treatment outcomes and identifying therapeutically actionable clones. The next frontier lies in enhancing model interpretability and cross-cohort generalizability to facilitate clinical integration.

### 5.3. Infectious Disease: Modeling Immune Dynamics and Cross-Protective Immunity

In the context of infectious disease, the BCR repertoire serves as a dynamic readout of the evolving immune response. The temporal and multi-phase nature of infection makes this field a natural fit for AI, particularly for tasks such as disease stratification, modeling cross-reactive immunity, and responding to emerging pathogens.

For real-time monitoring and stratification, AI models can capture subtle repertoire features with prognostic relevance. During the SARS-CoV-2 pandemic, features such as BCR clonal diversity, SHM distribution, and public clone frequencies were utilized to construct models of immune subtypes and disease severity. One study utilized random forest algorithms to distinguish mild from severe COVID-19 by incorporating SHM profiles and CDR3 length features, highlighting the utility of BCR-derived biomarkers for outcome prediction [72]. Transformer-based models have also been applied to sequence data longitudinally, identifying early clonal expansions linked to neutralizing antibody development [73].

In the search for cross-protective antibodies and pan-viral vaccines, AI helps identify conserved immune features across virus families. Similar to the cancer setting, researchers aim to uncover public neutralizing antibodies across viral infections. Neural network models analyzing repertoires from SARS-CoV-1, SARS-CoV-2, and MERS-CoV revealed conserved CDRH3 motifs capable of cross-virus binding, offering promising candidates for universal vaccine design [74]. Similarly, studies on dengue virus infection similarly used AI to map cross-serotype immune recognition and uncover conserved epitopes predictive of cross-protection [75]. Beyond motif discovery, large-scale repertoire modeling has also demonstrated potential for direct disease classification. A recent large-scale study applied machine learning approaches to B cell and T cell receptor repertoires across infectious and autoimmune conditions, achieving accurate disease stratification at the cohort level. Importantly, analyses restricted to BCR sequences alone retained substantial diagnostic signal, underscoring the translational feasibility of repertoire-based diagnostics independent of TCR information [76].

In the realm of vaccine response modeling and rapid-response preparedness, multimodal deep learning frameworks integrate sequence features, clonal dynamics, serology, and clinical outcomes to reconstruct personalized immune trajectories [77]. These models help explain diminished vaccine responses in the elderly and evaluate differential outcomes between primary and breakthrough infections. Structure-aware generative models are now being applied to small-sample pathogen settings, offering a route to computationally designing candidate antibodies during novel outbreaks [78].

In conclusion, AI extends BCR analysis in infectious diseases far beyond static sequence cataloging into temporal modeling of immune response, cross-reactivity mapping, and emergent threat preparedness.

### 5.4. Autoimmune Disease: Identifying Pathogenic Clones and Molecular Signatures

In autoimmune disease, the breakdown of B cell tolerance and the emergence of autoreactive clones are central to pathogenesis. AI-enhanced BCR analytics offer new lenses to examine these conditions across diagnostics, disease activity monitoring, and mechanistic insight.

At the level of diagnostic biomarkers, machine learning is adept at identifying subtle but discriminatory repertoire patterns. For instance, random forest classifiers trained on SHM distributions and CDR3 length profiles successfully distinguished Crohn’s disease patients from healthy controls, suggesting that BCR features can serve as noninvasive diagnostic tools in autoimmune settings [79]. In multiple sclerosis, lineage analysis using large-scale AIRR-seq data revealed correlations between clonal structure and disease subtypes, offering support for disease stratification and longitudinal monitoring [80].

For monitoring disease activity, deep learning models are integrating BCR sequence data, clonal expansion trends, and clinical scores. In diseases like systemic lupus erythematosus (SLE) and rheumatoid arthritis (RA), decreased diversity, altered SHM distributions, and increased public clone usage have been associated with active disease and relapse risk, enabling the construction of “immunologic activity indices” [81].

At the mechanistic level, BCR analysis can bridge genetic mutations to autoimmune phenotypes. In deficiency of adenosine deaminase 2 (DADA2), patients exhibit impaired B cell development and clonal structure, characterized by reduced SHM and more conservative repertoire composition, suggesting a failure in tolerance enforcement and expansion of autoreactive clones [82]. Similar analytic frameworks are being extended to allergic diseases to model IgE class-switch recombination and allergen-specific clone evolution, providing new opportunities for stratification and targeted interventions.

Collectively, AI is shifting autoimmune research from population-level associations to clone-level resolution, enabling the discovery of new biomarkers and providing mechanistic windows into tolerance disruption.

## 6. Current Bottlenecks and Challenges

Despite the substantial potential demonstrated by machine learning and deep learning approaches in BCR repertoire analysis, their broad deployment in large-scale, real-world datasets and clinical settings is still hampered by fundamental bottlenecks that cannot be addressed through computational scaling alone. These challenges primarily arise along four dimensions: data-level heterogeneity and lack of standardization, model-level limitations in interpretability and causal inference, immunological constraints in low-abundance but functionally important clones, and restricted generalizability across individuals and disease contexts. Failure to address these limitations risks relegating even high-performing models to “beautiful benchmarks”, rather than robust, reusable, and translatable immunological tools.

### 6.1. Data Standardization and Heterogeneity

Among the technical barriers, insufficient data standardization remains the most fundamental and persistent. BCR repertoire data are extremely sensitive to upstream experimental variables, such as sample collection methods, sequencing platforms, primer design, and sequencing depth, each of which can drastically affect observed clone composition and frequency [14,83]. Consequently, models performing well in one dataset often experience a sharp drop or even inversion of performance when applied to a different cohort, institution, or population. Clinical datasets further compound these challenges with imbalanced sample distributions and incomplete metadata undermining the external validity of AI models. In short, without “standardized and homogeneous” data foundations, any discussion of “generalizable” modeling remains largely theoretical.

### 6.2. Model Interpretability and the “Black-Box” Problem

The second bottleneck concerns interpretability. Deep models, particularly Transformers and multi-layer neural architectures, possess impressive representational power but lack transparent decision mechanisms, making it difficult to extract biologically verifiable explanations. For immunologists seeking to reverse-engineer mechanisms from model predictions, this opacity creates a wide gulf between a model being right and understanding why it is right. In clinical contexts, this challenge becomes even more acute. Clinicians and regulatory bodies demand clear evidence of which sequence features drive predictions and whether they align with known immunological pathways [84]. The absence of interpretability erodes trust, limits translational adoption, and weakens the model’s value as a hypothesis generator to guide experimental design.

Although a variety of post hoc interpretability methods have been proposed in recent years, their ability to provide true mechanistic insight remains limited [85]. In Transformer-based models, attention weights and intermediate representations can be analyzed to identify residues, CDR loops, or sequence patterns associated with model predictions [86]. In addition, perturbation-based strategies, such as residue masking, in silico mutagenesis, and controlled sequence variation, enable systematic evaluation of how input changes affect model outputs and internal activations [87]. However, these signals are often correlational rather than causal, and their correspondence to underlying biological mechanisms is neither stable nor reliable [88]. In many cases, attention weights or attribution scores do not consistently or reliably map to experimentally validated functional determinants. For example, in current BCR research, a common paradigm remains that computational analysis is followed by experimental validation of selected candidate genes or antibody sequences on a case-by-case basis. In this sense, computational models primarily function as pre-screening tools that filter out a subset of unlikely candidates, rather than definitively identifying functionally relevant ones. Even for the filtered-out candidates, the potential for false negatives persists, and whether they are truly irrelevant often remains uncertain. Bridging the gap between model interpretability and biological meaning therefore remains a central challenge, requiring tighter integration with structural analysis and experimental validation.

### 6.3. Functional Annotation and Causal Inference of Low-Abundance Clones

A third and particularly thorny challenge lies in modeling low-frequency clones, which expose a fundamental mismatch between biological relevance and statistical visibility. Although models perform robustly on high-abundance sequences, many functionally decisive B cell clones, including those mediating cross-reactivity, long-term protection, or breakdown of tolerance, are concentrated in the extreme tail of the abundance distribution [81]. Because these clones generate weak sequencing signals, they are often systematically down-weighted during model optimization.

Overcoming this bottleneck will require algorithmic frameworks that explicitly account for biological priors and rare-event sensitivity, including contextual information such as tissue origin, co-stimulatory signals, and the somatic microenvironment. Crucially, these computational advances must be coupled with experimental feedback loops to bridge the gap between in silico inference and empirical validation, and to restore causal interpretability to repertoire-level analyses.

### 6.4. Benchmarking and Evaluation Strategies in AI-Driven Immune Repertoire Analysis

Despite the rapid advancement of AI models in immune repertoire analysis, standardized benchmarking and evaluation strategies remain underdeveloped. One key challenge lies in the lack of consistent cross-cohort validation. Many models are trained and evaluated within a single dataset, which can lead to overestimation of performance due to cohort-specific biases. Recent studies emphasize the importance of validating models across independent cohorts to ensure robustness and generalizability across diverse populations and disease contexts [38].

In terms of evaluation metrics, antigen specificity prediction is typically assessed using classification-based metrics such as accuracy, precision, recall, F1-score, and area under the receiver operating characteristic curve (AUC-ROC) [89]. However, these metrics alone may not fully capture biological relevance, particularly in highly imbalanced datasets or when predicting rare antigen-specific clones. More nuanced evaluation strategies, including calibration metrics, precision–recall curves, and ranking-based measures, are increasingly being explored [90,91]. Furthermore, structural validation and experimental validation play a critical role in assessing model outputs. Structural validation may involve consistency with predicted or experimentally determined antibody–antigen complexes, using tools such as AlphaFold or molecular docking simulations. Experimental validation, including binding assays, neutralization tests, or functional screening, remains the gold standard for confirming model predictions. The integration of these validation layers is essential for translating computational predictions into biologically and clinically meaningful insights.

Collectively, these considerations highlight the urgent need for standardized benchmarking frameworks, curated reference datasets, and reproducible evaluation protocols to enable fair comparison across models and accelerate progress in AI-driven immune repertoire analysis.

### 6.5. Cross-Individual and Cross-Disease Generalizability

The fourth major challenge concerns the transferability of models across individuals, cohorts, and disease contexts. Human BCR repertoires are shaped by diverse genetic, environmental, and clinical variables, including HLA background, immune history, age, and disease stage, yielding a high degree of individuality [92]. Consequently, many AI models learn context-specific correlations rather than causal, generalizable immunological principles, leading to performance drift when applied beyond the training population.

To make AI genuinely useful for personalized immune profiling and therapeutic simulation, future models must embrace transfer learning, multi-task learning, and causal inference frameworks. These approaches can explicitly model population structure and disease heterogeneity while leveraging cross-cohort, cross-species, and cross-tissue datasets to disentangle context-dependent from conserved immunological rules. Only through such methodological and theoretical expansion can AI move beyond cohort-specific optimization toward reusable, foundational immune modeling frameworks.

## 7. Towards a Closed-Loop Immune Modeling System

To transition from passive pattern discovery to proactive immune intervention, the field must pursue an integrated, closed-loop immune modeling system. This system synthesizes data acquisition, sequence analysis, functional prediction, generative design, experimental validation, and model refinement into a continuous feedback cycle. The ultimate objective extends beyond merely explaining immune phenomena; it aims to predict and rationally design desired immune responses. Realizing this vision necessitates coordinated breakthroughs in data infrastructure, model interpretability, and generative capabilities.

### 7.1. The Foundation of the Loop: Building a Standardized Multimodal Data Ecosystem

The cornerstone of such a closed-loop system is high-quality, standardized, multimodal data. Current immunorepertoire landscapes remain fragmented with significant disparities in format, annotation, and metadata quality that constrain cross-cohort generalization. Establishing robust, interoperable data-sharing platforms is thus the first step toward a truly closed-loop ecosystem.

Recent initiatives have begun to lay this groundwork. Public repositories such as SRA, GEO, and ENA [93,94,95], along with specialized commercial resources like ImmuneACCESS [96], provide large-scale repositories for raw sequencing data. Foundational databases such as IMGT [97] and integrative platforms like iReceptor [98], OAS [99], huARdb [100], PIRD [101], ImmuneDB [102], VDJdb [103], and IEDB [104] collectively offer complementary information spanning V(D)J annotations, clonal structures, antigen–antibody interactions, and validated epitopes. Commercial resources such as TABS and SAbDab-therapeutic further enrich this landscape with structural and clinical data critical for antibody development [105].

Pathogen-specific curated databases have also played a critical role in advancing BCR modeling. For example, CoV-AbDab [106] represents the largest curated repository of SARS-CoV-2-binding antibodies and has served as a primary source of positive labels in numerous predictive modeling studies. However, current pathogen-focused BCR datasets exhibit significant limitations, as they are heavily concentrated on SARS-CoV-2 and, to a lesser extent, HIV. Moreover, much of the available SARS-CoV-2 data is derived from antibodies targeting the original wild-type strain rather than variants that predominated during later stages of the pandemic. This imbalance introduces substantial bias, affecting not only the statistical characterization of antigen-specific B cell responses but also the training of antibody–antigen interaction models, thereby limiting their generalizability and practical applicability. To address these challenges, multiple research groups are actively pursuing strategies to diversify training datasets and mitigate antigen-specific bias through alternative data acquisition and modeling approaches.

The above databases are summarized in Table 3. Together, these efforts provide the scaffolding for a standardized, data-rich ecosystem, upon which the next generation of intelligent immune modeling will be built.

Beyond data standardization, the system requires cross-modality connectivity—fusing molecular, structural, and clinical dimensions into a unified analytical framework. The next frontier lies in the integration of BCR sequences, clonal phylogenies, antigen 3D structures, single-cell transcriptomes, and clinical phenotypes [107]. Such multimodal fusion serves a dual purpose: first, it enriches the biological context, enabling fine-grained reconstruction of B cell developmental trajectories and functional heterogeneity; second, it promises to substantially enhance model generalizability across diseases and populations, overcoming the fragmentation and incompleteness inherent in traditional single-modality analyses [108].

While substantial progress has been made in data integration, translating this multimodal foundation into a fully functional closed-loop system remains a significant challenge. Nevertheless, several recent studies have begun to approximate components of this paradigm. For example, deep learning-driven antibody design frameworks have been coupled with high-throughput experimental screening, enabling iterative cycles of sequence generation, functional evaluation, and model refinement [109]. Similarly, workflows integrating single-cell BCR sequencing with antigen specificity prediction and subsequent experimental validation (e.g., binding or neutralization assays) represent early forms of feedback-driven immune modeling [23,110]. These efforts suggest that key elements of the closed-loop system are already emerging within current experimental and computational infrastructures.

From a practical perspective, implementing such a closed-loop system is increasingly feasible through the modular integration of computational and experimental pipelines. A realistic workflow may involve: (i) large-scale acquisition of BCR repertoires via bulk or single-cell sequencing; (ii) representation learning using antibody language models or other deep architectures; (iii) prediction of antigen specificity, affinity, or clonal expansion; (iv) structural modeling and in silico validation using tools such as AlphaFold and molecular docking [111]; (v) experimental validation through binding assays or functional screening; and (vi) iterative model updating based on experimental feedback, where validation results (e.g., failed binders) are used as negative training labels to refine predictive accuracy [112]. This stepwise integration bridges data generation, modeling, and validation into a continuous optimization loop, transforming the closed-loop framework from a conceptual vision into a progressively realizable system.

### 7.2. The Core Engine of the Loop: From “Black Box” to Explainable AI

With a solid data foundation established, the heart of the closed-loop system lies in developing an intelligent engine that understands rather than merely fits the data. Future progress will depend on balancing predictive performance with interpretability. The deep incorporation of explainable artificial intelligence (XAI) tools provides a key pathway toward this balance.

Techniques such as attention visualization, feature attribution analysis, and counterfactual reasoning allow models to explicitly highlight which amino acid residues, CDRs, structural domains, or clonal branches are decisive for prediction [60,113,114,115]. This interpretive layer fosters trust and translates computational outputs into testable biological hypotheses, thereby guiding experimental validation. For example, in a GNN modeling B cell clonal evolution, an interpretable framework can pinpoint specific mutations along a lineage that drive tolerance breakdown or broad neutralization. This transition reflects the maturation of AI from a black-box predictor to a mechanism-driven, hypothesis-generating engine for scientific inquiry.

### 7.3. The Productive Output of the Loop: Generative AI for Intelligent Antibody and Vaccine Design

Once immune principles are deeply learned, the ultimate value of a closed-loop system lies not merely in explaining existing data but in designing immunologically valuable solutions yet to exist. The advent of generative AI is transforming this vision into reality. Emerging architectures, such as VAEs and diffusion models, effectively internalize the structural and evolutionary grammar of the immune system, enabling de novo design of antibodies or immunogens with specified functions [54,58]. This paradigm represents a fundamental transition in BCR analysis from passive discovery to active design, offering a computational pathway toward accelerated antibody therapeutics and next-generation vaccine development. It is, arguably, the most exciting and tangible manifestation of the closed-loop concept.

Taken together, the future of BCR repertoire analysis is evolving beyond isolated computational methods toward an integrated, closed-loop ecosystem, anchored in standardized multimodal data, empowered by interpretable models, and driven by generative design. In such a system, every model prediction or molecular design feeds directly into experimental validation, while every experimental result returns to enrich the data ecosystem, fueling the next round of model refinement and hypothesis generation. This continuous cycle of analysis, prediction, design, validation, and optimization promises to bridge the long-standing divide between theoretical modeling and clinical application. Ultimately, it will transform BCR research from a descriptive science into an intervention-ready, design-oriented discipline, inaugurating a new era of intelligence-driven precision immunology.

## 8. Conclusions

From a methodological perspective, AI-driven BCR repertoire analysis can be viewed as a multiscale hierarchy of models, ranging from CNNs capturing local motifs and RNNs modeling sequential dynamics to Transformer-based language models learning global sequence semantics and GNNs reconstructing clonal topology. Structure-aware and generative models further extend this framework toward three-dimensional inference and antibody design, highlighting the complementary roles of different architectures across biological scales.

Artificial intelligence is redefining how we decode and harness the human B cell repertoire. Beyond serving as a computational microscope for complex immune data, AI is gradually constructing a closed-loop framework that unites sequence, structure, function, and clinical phenotype. While challenges in data standardization and validation persist, the accelerating convergence of multimodal learning, XAI, and generative modeling, supported by high-quality community databases, warrants strong optimism. In the coming decade, intelligent BCR analysis is poised to become a core engine of precision immunology, powering advances in diagnostic stratification, personalized vaccine development, and next-generation antibody therapeutics, and propelling the field toward a data-driven, intelligently designed future.

## Figures and Tables

**Table 1:** Representative machine learning and deep learning architectures for BCR repertoire analysis.

Model Type	Representative Architectures	Advantages	Limitations	Typical Applications
CNN	ConvNet, ResNet	Efficiently identifies local sequence motifs and short-range patterns; well-suited for capturing CDR-local structural signals	Limited in modeling long-range dependencies; lacks holistic sequence semantics	CDR motif recognition; local-feature-driven receptor classification
RNN	LSTM, GRU	Captures sequential dependencies and mutation trajectories; suitable for modeling continuous mutational paths	Difficult to parallelize; unstable training; prone to gradient vanishing	Modeling somatic mutation paths; analysis of antigen-binding regions
Transformer	BERT, ESM, IgLM, AntiBERTa	Strong capability for global context modeling; self-supervised pretraining enables learning of “immunological semantics”; state of the art in sequence embedding	Large number of parameters; high computational cost; requires extensive pretraining data	Antigen specificity prediction; epitope mapping; functional annotation; multimodal integration
GNN	GCN, GAT, GraphSAGE	Models the graph topology of SHM networks and clonal lineages; captures complex interactions among mutations	Requires explicit graph construction; complex training; sensitive to data quality	SHM trajectory inference; clonal family reconstruction; clonal network analysis
VAE/GAN	VAE, Diffusion Model	Learns latent distributions of the sequence–structure space; supports antibody sequence generation and immune response simulation	Limited ability to incorporate structural constraints; weak target controllability	Antibody design; affinity maturation simulation; immunogen engineering

Abbreviations: CNN, convolutional neural network; CDR, complementarity-determining region; RNN, recurrent neural network; LSTM, long short-term memory; GRU, gated recurrent unit; BERT, bidirectional encoder representations from transformers; ESM, evolutionary scale modeling; IgLM, immunoglobulin language model; GNN, graph neural network; GCN, graph convolutional network; GAT, graph attention network; SHM, somatic hypermutation; VAE, variational autoencoder; GAN, generative adversarial network.

**Table 3:** Comprehensive overview of AIRR-Seq databases for BCR/TCR data analysis.

Database Name	Notes/Description	URL	Reference
SRA/GEO/ENA	The three major repositories for raw sequencing data, containing extensive AIRR-seq datasets. Support keyword search and bulk download of BCR/IgH sequences	https://www.ncbi.nlm.nih.gov/sra (accessed on 1 November 2025) https://www.ncbi.nlm.nih.gov/geo/ (accessed on 1 November 2025) https://www.ebi.ac.uk/ena/browser/home (accessed on 1 November 2025)	[93,94,95]
ImmuneACCESS (Adaptive Biotechnologies)	A commercial platform offering partially open-access BCR/TCR datasets with visualization and clonotype frequency analysis tools, enabling interactive exploration	https://clients.adaptivebiotech.com/immuneaccess (accessed on 1 November 2025)	[96]
IMGT (International ImMunoGeneTics)	The most authoritative global resource for immunogenetic information, supporting V/D/J gene annotation, allele comparison, and BCR structural modeling	https://www.imgt.org (accessed on 1 November 2025)	[97]
iReceptor	Enables cross-platform query, analysis, and download of AIRR data from multiple projects with unified data standards, facilitating standardized comparative studies	https://gateway.ireceptor.org (accessed on 1 November 2025)	[98]
OAS (Observed Antibody Space)	A public database containing over one billion BCR/antibody sequences, suitable for modeling and training deep learning algorithms	http://opig.stats.ox.ac.uk/webapps/oas/ (accessed on 1 November 2025)	[99]
huARdb	Integrates human single-cell transcriptomic data with antigen receptor clonotypes, supporting analysis of cell subtypes, lineages, and transcriptional states	https://huarc.net/v2/database/ (accessed on 1 November 2025)	[100]
PIRD (CNGB)	Collects AIRR data from humans and other vertebrates under various phenotypic and disease conditions; provides both raw and annotated data for download	https://db.cngb.org/pird/home/ (accessed on 1 November 2025)	[101]
ImmuneDB	Functions as both a database and an analytical framework, supporting large-scale AIRR data annotation, clonal clustering, and frequency analysis	https://immunedb.readthedocs.io/en/latest/ (accessed on 1 November 2025)	[102]
VDJdb	Focuses on TCR (and partially BCR) sequences with known antigen specificities, enabling target inference and model validation	https://vdjdb.cdr3.net (accessed on 1 November 2025)	[103]
IEDB (Immune Epitope Database)	Contains experimentally validated B and T cell epitopes across viruses, bacteria, and autoimmune contexts, supporting cross-species comparison	https://www.iedb.org (accessed on 1 November 2025)	[104]
TABS (commercial use)	A commercial antibody database integrating information on therapeutic antibody development, including sequences, affinities, and indications (registration required)	https://tabs.craic.com/users/sign_in (accessed on 1 November 2025)	N/A
SAbDab-therapeutic antibodies	Curates structural, sequence, and indication data for approved and clinical-stage therapeutic antibodies, suitable for structure-based modeling and drug design	https://opig.stats.ox.ac.uk/webapps/sabdab-sabpred/therasabdab/search/ (accessed on 1 November 2025)	[105]
CoV-AbDab	The largest single database of BCR sequences that bind to a specific pathogen. It has been widely utilized in countless studies on the BCR sequences of COVID-19 patients	https://opig.stats.ox.ac.uk/webapps/covabdab/ (accessed on 1 November 2025)	[106]

Abbreviations: AIRR-seq, adaptive immune receptor repertoire sequencing; BCR, B cell receptor; TCR, T cell receptor; IgH, immunoglobulin heavy chain; SRA, Sequence Read Archive; GEO, Gene Expression Omnibus; ENA, European Nucleotide Archive; IMGT, the international ImMunoGeneTics information system; OAS, Observed Antibody Space; PIRD, Pan Immune Repertoire Database; IEDB, Immune Epitope Database.

## Data Availability

No new data were created or analyzed in this study. Data sharing is not applicable to this article.

## Abbreviations

The following abbreviations are used in this manuscript:

BCR	B cell receptor
AIRR-seq	Adaptive immune receptor repertoire sequencing
AI	Artificial intelligence
GNN	Graph neural network
SHM	Somatic hypermutation
CDR	Complementarity-determining region
UMI	Unique molecular identifier
CNN	Convolutional neural network
RNN	Recurrent neural network
LSTM	Long short-term memory
GRU	Gated recurrent unit
AbLM	Antibody language model
VAE	Variational autoencoder
GAN	Generative adversarial network
TME	Tumor microenvironment
TIL-B	Tumor-infiltrating B cell
NLP	Natural language processing
GCN	Graph convolutional network
SRA	Sequence Read Archive
GEO	Gene Expression Omnibus
ENA	European Nucleotide Archive
IMGT	International ImMunoGeneTics information system
IEDB	Immune Epitope Database
OAS	Observed Antibody Space
XAI	Explainable artificial intelligence
DADA2	Adenosine deaminase 2 deficiency
SLE	Systemic lupus erythematosus
RA	Rheumatoid arthritis
PD-1	Programmed cell death protein 1
PD-L1	Programmed death-ligand 1
HIV	Human immunodeficiency virus
SARS-CoV-2	Severe acute respiratory syndrome coronavirus 2
MERS-CoV	Middle East respiratory syndrome coronavirus

## References

[1] Jordan M.I., Mitchell T.M. (2015). Machine Learning: Trends, Perspectives, and Prospects. Science.

[2] Butler K.T., Davies D.W., Cartwright H., Isayev O., Walsh A. (2018). Machine Learning for Molecular and Materials Science. Nature.

[3] Schneider G. (2018). Automating Drug Discovery. Nat. Rev. Drug Discov..

[4] Raccuglia P., Elbert K.C., Adler P.D.F., Falk C., Wenny M.B., Mollo A., Zeller M., Friedler S.A., Schrier J., Norquist A.J. (2016). Machine-Learning-Assisted Materials Discovery Using Failed Experiments. Nature.

[5] Patel K.K., Kar A., Jha S.N., Khan M.A. (2012). Machine Vision System: A Tool for Quality Inspection of Food and Agricultural Products. J. Food Sci. Technol..

[6] Tonegawa S. (1983). Somatic Generation of Antibody Diversity. Nature.

[7] McKean D., Huppi K., Bell M., Staudt L., Gerhard W., Weigert M. (1984). Generation of Antibody Diversity in the Immune Response of BALB/c Mice to Influenza Virus Hemagglutinin. Proc. Natl. Acad. Sci. USA.

[8] Hershberg U., Luning Prak E.T. (2015). The Analysis of Clonal Expansions in Normal and Autoimmune B Cell Repertoires. Philos. Trans. R. Soc. Lond. B Biol. Sci..

[9] Elhanati Y., Sethna Z., Marcou Q., Callan C.G., Mora T., Walczak A.M. (2015). Inferring Processes Underlying B-Cell Repertoire Diversity. Philos. Trans. R. Soc. Lond. B Biol. Sci..

[10] Rappazzo C.G., Fernández-Quintero M.L., Mayer A., Wu N.C., Greiff V., Guthmiller J.J. (2023). Defining and Studying B Cell Receptor and TCR Interactions. J. Immunol..

[11] Reth M., Kläsener K., Nitschke L., Gold M.R. (2024). Structure and Signalling Function of the B-Cell Antigen Receptor and Its Coreceptors. Molecular Biology of B Cells.

[12] Friedensohn S., Khan T.A., Reddy S.T. (2017). Advanced Methodologies in High-Throughput Sequencing of Immune Repertoires. Trends Biotechnol..

[13] Vázquez Bernat N., Corcoran M., Hardt U., Kaduk M., Phad G.E., Martin M., Karlsson Hedestam G.B. (2019). High-Quality Library Preparation for NGS-Based Immunoglobulin Germline Gene Inference and Repertoire Expression Analysis. Front. Immunol..

[14] Trück J., Eugster A., Barennes P., Tipton C.M., Luning Prak E.T., Bagnara D., Soto C., Sherkow J.S., Payne A.S., Lefranc M.-P. (2021). Biological Controls for Standardization and Interpretation of Adaptive Immune Receptor Repertoire Profiling. eLife.

[15] Eugster A., Bostick M.L., Gupta N., Mariotti-Ferrandiz E., Kraus G., Meng W., Soto C., Trück J., Stervbo U., Luning Prak E.T. (2022). AIRR Community Guide to Planning and Performing AIRR-Seq Experiments. Methods Mol. Biol..

[16] Setliff I., McDonnell W.J., Raju N., Bombardi R.G., Murji A.A., Scheepers C., Ziki R., Mynhardt C., Shepherd B.E., Mamchak A.A. (2018). Multi-Donor Longitudinal Antibody Repertoire Sequencing Reveals the Existence of Public Antibody Clonotypes in HIV-1 Infection. Cell Host Microbe.

[17] Galson J.D., Trück J., Fowler A., Clutterbuck E.A., Münz M., Cerundolo V., Reinhard C., van der Most R., Pollard A.J., Lunter G. (2015). Analysis of B Cell Repertoire Dynamics Following Hepatitis B Vaccination in Humans, and Enrichment of Vaccine-Specific Antibody Sequences. eBioMedicine.

[18] Briney B., Inderbitzin A., Joyce C., Burton D.R. (2019). Commonality despite Exceptional Diversity in the Baseline Human Antibody Repertoire. Nature.

[19] Lee J., Paparoditis P., Horton A.P., Frühwirth A., McDaniel J.R., Jung J., Boutz D.R., Hussein D.A., Tanno Y., Pappas L. (2019). Persistent Antibody Clonotypes Dominate the Serum Response to Influenza Over Multiple Years and Repeated Vaccinations. Cell Host Microbe.

[20] Bournazos S., Vo H.T.M., Duong V., Auerswald H., Ly S., Sakuntabhai A., Dussart P., Cantaert T., Ravetch J.V. (2021). Antibody Fucosylation Predicts Disease Severity in Secondary Dengue Infection. Science.

[21] Emerson R.O., DeWitt W.S., Vignali M., Gravley J., Hu J.K., Osborne E.J., Desmarais C., Klinger M., Carlson C.S., Hansen J.A. (2017). Immunosequencing Identifies Signatures of Cytomegalovirus Exposure History and HLA-Mediated Effects on the T Cell Repertoire. Nat. Genet..

[22] Cowell L.G. (2020). The Diagnostic, Prognostic, and Therapeutic Potential of Adaptive Immune Receptor Repertoire Profiling in Cancer. Cancer Res..

[23] Mason D.M., Friedensohn S., Weber C.R., Jordi C., Wagner B., Meng S.M., Ehling R.A., Bonati L., Dahinden J., Gainza P. (2021). Optimization of Therapeutic Antibodies by Predicting Antigen Specificity from Antibody Sequence via Deep Learning. Nat. Biomed. Eng..

[24] Schuster S.J., Bishop M.R., Tam C.S., Waller E.K., Borchmann P., McGuirk J.P., Jäger U., Jaglowski S., Andreadis C., Westin J.R. (2019). Tisagenlecleucel in Adult Relapsed or Refractory Diffuse Large B-Cell Lymphoma. N. Engl. J. Med..

[25] Boyd S.D., Marshall E.L., Merker J.D., Maniar J.M., Zhang L.N., Sahaf B., Jones C.D., Simen B.B., Hanczaruk B., Nguyen K.D. (2009). Measurement and Clinical Monitoring of Human Lymphocyte Clonality by Massively Parallel VDJ Pyrosequencing. Sci. Transl. Med..

[26] Weinstein J.A., Jiang N., White R.A., Fisher D.S., Quake S.R. (2009). High-Throughput Sequencing of the Zebrafish Antibody Repertoire. Science.

[27] DeWitt W.S., Lindau P., Snyder T.M., Sherwood A.M., Vignali M., Carlson C.S., Greenberg P.D., Duerkopp N., Emerson R.O., Robins H.S. (2016). A Public Database of Memory and Naive B-Cell Receptor Sequences. PLoS ONE.

[28] Greiff V., Menzel U., Miho E., Weber C., Riedel R., Cook S., Valai A., Lopes T., Radbruch A., Winkler T.H. (2017). Systems Analysis Reveals High Genetic and Antigen-Driven Predetermination of Antibody Repertoires throughout B Cell Development. Cell Rep..

[29] Sela-Culang I., Kunik V., Ofran Y. (2013). The Structural Basis of Antibody-Antigen Recognition. Front. Immunol..

[30] Richardson E., Galson J.D., Kellam P., Kelly D.F., Smith S.E., Palser A., Watson S., Deane C.M. (2021). A Computational Method for Immune Repertoire Mining That Identifies Novel Binders from Different Clonotypes, Demonstrated by Identifying Anti-Pertussis Toxoid Antibodies. MAbs.

[31] Imkeller K., Wardemann H. (2018). Assessing Human B Cell Repertoire Diversity and Convergence. Immunol. Rev..

[32] Wong W.K., Robinson S.A., Bujotzek A., Georges G., Lewis A.P., Shi J., Snowden J., Taddese B., Deane C.M. (2021). Ab-Ligity: Identifying Sequence-Dissimilar Antibodies That Bind to the Same Epitope. MAbs.

[33] Rubelt F., Bolen C.R., McGuire H.M., Vander Heiden J.A., Gadala-Maria D., Levin M., Euskirchen G.M., Mamedov M.R., Swan G.E., Dekker C.L. (2016). Individual Heritable Differences Result in Unique Cell Lymphocyte Receptor Repertoires of Naïve and Antigen-Experienced Cells. Nat. Commun..

[34] Patin E., Hasan M., Bergstedt J., Rouilly V., Libri V., Urrutia A., Alanio C., Scepanovic P., Hammer C., Jönsson F. (2018). Natural Variation in the Parameters of Innate Immune Cells Is Preferentially Driven by Genetic Factors. Nat. Immunol..

[35] Hoehn K.B., Kleinstein S.H. (2024). B Cell Phylogenetics in the Single Cell Era. Trends Immunol..

[36] Collins A.M., Jackson K.J.L. (2018). On Being the Right Size: Antibody Repertoire Formation in the Mouse and Human. Immunogenetics.

[37] Six A., Mariotti-Ferrandiz M.E., Chaara W., Magadan S., Pham H.-P., Lefranc M.-P., Mora T., Thomas-Vaslin V., Walczak A.M., Boudinot P. (2013). The Past, Present, and Future of Immune Repertoire Biology—The Rise of Next-Generation Repertoire Analysis. Front. Immunol..

[38] Pavlović M., Scheffer L., Motwani K., Kanduri C., Kompova R., Vazov N., Waagan K., Bernal F.L.M., Costa A.A., Corrie B. (2021). The immuneML Ecosystem for Machine Learning Analysis of Adaptive Immune Receptor Repertoires. Nat. Mach. Intell..

[39] Tang C., Krantsevich A., MacCarthy T. (2022). Deep Learning Model of Somatic Hypermutation Reveals Importance of Sequence Context beyond Hotspot Targeting. iScience.

[40] Johnson M.M., Sung K., Haddox H.K., Vora A.A., Araki T., Victora G.D., Song Y.S., Fukuyama J., Matsen F.A. (2025). Nucleotide Context Models Outperform Protein Language Models for Predicting Antibody Affinity Maturation. PLoS Comput. Biol..

[41] Widrich M., Schäfl B., Pavlović M., Ramsauer H., Gruber L., Holzleitner M., Brandstetter J., Sandve G.K., Greiff V., Hochreiter S. (2020). Modern Hopfield Networks and Attention for Immune Repertoire Classification. Proceedings of the 34th International Conference on Neural Information Processing Systems.

[42] Lee H., Shin K., Lee Y., Lee S., Lee S., Lee E., Kim S.W., Shin H.Y., Kim J.H., Chung J. (2024). Identification of B Cell Subsets Based on Antigen Receptor Sequences Using Deep Learning. Front. Immunol..

[43] Saka K., Kakuzaki T., Metsugi S., Kashiwagi D., Yoshida K., Wada M., Tsunoda H., Teramoto R. (2021). Antibody Design Using LSTM Based Deep Generative Model from Phage Display Library for Affinity Maturation. Sci. Rep..

[44] Ostrovsky-Berman M., Frankel B., Polak P., Yaari G. (2021). Immune2vec: Embedding B/T Cell Receptor Sequences in ℝ N Using Natural Language Processing. Front. Immunol..

[45] Leem J., Mitchell L.S., Farmery J.H.R., Barton J., Galson J.D. (2022). Deciphering the Language of Antibodies Using Self-Supervised Learning. Patterns.

[46] Choi S., Kim D. (2024). B Cell Epitope Prediction by Capturing Spatial Clustering Property of the Epitopes Using Graph Attention Network. Sci. Rep..

[47] Lee Y., Lee H., Shin K., Kwon S. (2023). GRIP: Graph Representation of Immune Repertoire Using Graph Neural Network and Transformer. Proc. AAAI Conf. Artif. Intell..

[48] Abramson J., Adler J., Dunger J., Evans R., Green T., Pritzel A., Ronneberger O., Willmore L., Ballard A.J., Bambrick J. (2024). Accurate Structure Prediction of Biomolecular Interactions with AlphaFold 3. Nature.

[49] Eguchi R.R., Choe C.A., Huang P.-S. (2022). Ig-VAE: Generative Modeling of Protein Structure by Direct 3D Coordinate Generation. PLoS Comput. Biol..

[50] Vu M.H., Akbar R., Robert P.A., Swiatczak B., Sandve G.K., Greiff V., Haug D.T.T. (2023). Linguistically Inspired Roadmap for Building Biologically Reliable Protein Language Models. Nat. Mach. Intell..

[51] Watson J.L., Juergens D., Bennett N.R., Trippe B.L., Yim J., Eisenach H.E., Ahern W., Borst A.J., Ragotte R.J., Milles L.F. (2023). De Novo Design of Protein Structure and Function with RFdiffusion. Nature.

[52] Hie B.L., Shanker V.R., Xu D., Bruun T.U.J., Weidenbacher P.A., Tang S., Wu W., Pak J.E., Kim P.S. (2024). Efficient Evolution of Human Antibodies from General Protein Language Models. Nat. Biotechnol..

[53] Castro K.M., Watson J.L., Wang J., Southern J., Ayardulabi R., Georgeon S., Rosset S., Baker D., Correia B.E. (2026). Accurate Single-Domain Scaffolding of Three Nonoverlapping Protein Epitopes Using Deep Learning. Nat. Chem. Biol..

[54] Akbar R., Robert P.A., Weber C.R., Widrich M., Frank R., Pavlović M., Scheffer L., Chernigovskaya M., Snapkov I., Slabodkin A. (2022). In Silico Proof of Principle of Machine Learning-Based Antibody Design at Unconstrained Scale. MAbs.

[55] Rives A., Meier J., Sercu T., Goyal S., Lin Z., Liu J., Guo D., Ott M., Zitnick C.L., Ma J. (2021). Biological Structure and Function Emerge from Scaling Unsupervised Learning to 250 Million Protein Sequences. Proc. Natl. Acad. Sci. USA.

[56] Olsen T.H., Moal I.H., Deane C.M. (2022). AbLang: An Antibody Language Model for Completing Antibody Sequences. Bioinform. Adv..

[57] Choi Y. (2022). Artificial Intelligence for Antibody Reading Comprehension: AntiBERTa. Patterns.

[58] Shuai R.W., Ruffolo J.A., Gray J.J. (2023). IgLM: Infilling Language Modeling for Antibody Sequence Design. Cell Syst..

[59] Wang M., Patsenker J., Li H., Kluger Y., Kleinstein S.H. (2025). Supervised Fine-Tuning of Pre-Trained Antibody Language Models Improves Antigen Specificity Prediction. PLoS Comput. Biol..

[60] Wang Y., Lv H., Teo Q.W., Lei R., Gopal A.B., Ouyang W.O., Yeung Y.-H., Tan T.J.C., Choi D., Shen I.R. (2024). An Explainable Language Model for Antibody Specificity Prediction Using Curated Influenza Hemagglutinin Antibodies. Immunity.

[61] Swanson O., Martin Beem J.S., Rhodes B., Wang A., Barr M., Chen H., Parks R., Saunders K.O., Haynes B.F., Wiehe K. (2023). Identification of CDRH3 Loops in the B Cell Receptor Repertoire That Can Be Engaged by Candidate Immunogens. PLoS Pathog..

[62] Mukhtar M., Wajeeha A.W., Zaidi N.U.S.S., Bibi N. (2022). Engineering Modified mRNA-Based Vaccine against Dengue Virus Using Computational and Reverse Vaccinology Approaches. Int. J. Mol. Sci..

[63] Khan A., Ammar Zahid M., Farrukh F., Salah Abdelsalam S., Mohammad A., Al-Zoubi R.M., Shkoor M., Ait Hssain A., Wei D.-Q., Agouni A. (2024). Integrated Structural Proteomics and Machine Learning-Guided Mapping of a Highly Protective Precision Vaccine against Mycoplasma Pulmonis. Int. Immunopharmacol..

[64] Norman R.A., Ambrosetti F., Bonvin A.M.J.J., Colwell L.J., Kelm S., Kumar S., Krawczyk K. (2020). Computational Approaches to Therapeutic Antibody Design: Established Methods and Emerging Trends. Brief. Bioinform..

[65] Xu X., Xu T., Zhou J., Liao X., Zhang R., Wang Y., Zhang L., Gao X. (2023). AB-Gen: Antibody Library Design with Generative Pre-Trained Transformer and Deep Reinforcement Learning. Genom. Proteom. Bioinform..

[66] He H., He B., Guan L., Zhao Y., Jiang F., Chen G., Zhu Q., Chen C.Y.-C., Li T., Yao J. (2024). De Novo Generation of SARS-CoV-2 Antibody CDRH3 with a Pre-Trained Generative Large Language Model. Nat. Commun..

[67] Konishi H., Komura D., Katoh H., Atsumi S., Koda H., Yamamoto A., Seto Y., Fukayama M., Yamaguchi R., Imoto S. (2019). Capturing the Differences between Humoral Immunity in the Normal and Tumor Environments from Repertoire-Seq of B-Cell Receptors Using Supervised Machine Learning. BMC Bioinform..

[68] Schmidt-Barbo P., Kalweit G., Naouar M., Paschold L., Willscher E., Schultheiß C., Märkl B., Dirnhofer S., Tzankov A., Binder M. (2024). Detection of Disease-Specific Signatures in B Cell Repertoires of Lymphomas Using Machine Learning. PLoS Comput. Biol..

[69] Selitsky S.R., Mose L.E., Smith C.C., Chai S., Hoadley K.A., Dittmer D.P., Moschos S.J., Parker J.S., Vincent B.G. (2019). Prognostic Value of B Cells in Cutaneous Melanoma. Genome Med..

[70] Che Y., Lee J., Abou-Taleb F., Rieger K.E., Satpathy A.T., Chang A.L.S., Chang H.Y. (2025). Induced B Cell Receptor Diversity Predicts PD-1 Blockade Immunotherapy Response. Proc. Natl. Acad. Sci. USA.

[71] Song B., Wang K., Na S., Yao J., Fattah F.J., Martin A.L., von Itzstein M.S., Yang D.M., Liu J., Xue Y. (2025). Profiling Antigen-Binding Affinity of B Cell Repertoires in Tumors by Deep Learning Predicts Immune-Checkpoint Inhibitor Treatment Outcomes. Nat. Cancer.

[72] Safra M., Tamari Z., Polak P., Shiber S., Matan M., Karameh H., Helviz Y., Levy-Barda A., Yahalom V., Peretz A. (2023). Altered Somatic Hypermutation Patterns in COVID-19 Patients Classifies Disease Severity. Front. Immunol..

[73] Richardson E., Willemsen L., Shinde P., Nielsen M., Peters B. (2025). Is the Vaccination-Induced B Cell Receptor Repertoire Predictable?. Immunoinformatics.

[74] Hie B., Zhong E.D., Berger B., Bryson B. (2021). Learning the Language of Viral Evolution and Escape. Science.

[75] Natsrita P., Charoenkwan P., Shoombuatong W., Mahalapbutr P., Faksri K., Chareonsudjai S., Rungrotmongkol T., Pipattanaboon C. (2024). Machine-Learning-Assisted High-Throughput Identification of Potent and Stable Neutralizing Antibodies against All Four Dengue Virus Serotypes. Sci. Rep..

[76] Zaslavsky M.E., Craig E., Michuda J.K., Sehgal N., Ram-Mohan N., Lee J.-Y., Nguyen K.D., Hoh R.A., Pham T.D., Röltgen K. (2025). Disease Diagnostics Using Machine Learning of B Cell and T Cell Receptor Sequences. Science.

[77] Wang Z., Lorenzi J.C.C., Muecksch F., Finkin S., Viant C., Gaebler C., Cipolla M., Hoffmann H.-H., Oliveira T.Y., Oren D.A. (2021). Enhanced SARS-CoV-2 Neutralization by Dimeric IgA. Sci. Transl. Med..

[78] Weber L.L., Reiman D., Roddur M.S., Qi Y., El-Kebir M., Khan A.A. (2024). Isotype-Aware Inference of B Cell Clonal Lineage Trees from Single-Cell Sequencing Data. Cell Genom..

[79] Safra M., Werner L., Peres A., Polak P., Salamon N., Schvimer M., Weiss B., Barshack I., Shouval D.S., Yaari G. (2023). A Somatic Hypermutation-Based Machine Learning Model Stratifies Individuals with Crohn’s Disease and Controls. Genome Res..

[80] Lomakin Y.A., Zvyagin I.V., Ovchinnikova L.A., Kabilov M.R., Staroverov D.B., Mikelov A., Tupikin A.E., Zakharova M.Y., Bykova N.A., Mukhina V.S. (2022). Deconvolution of B Cell Receptor Repertoire in Multiple Sclerosis Patients Revealed a Delay in tBreg Maturation. Front. Immunol..

[81] Bashford-Rogers R.J.M., Bergamaschi L., McKinney E.F., Pombal D.C., Mescia F., Lee J.C., Thomas D.C., Flint S.M., Kellam P., Jayne D.R.W. (2019). Analysis of the B Cell Receptor Repertoire in Six Immune-Mediated Diseases. Nature.

[82] Schultheiß C., Schmidt-Barbo P., Paschold L., Esperanzate C., Behn A., Mikolajczyk R., Kastner D.L., Aksentijevich I., Binder M. (2025). Deficiency of Adenosine Deaminase 2 Skews Adaptive Immune Repertoires toward Specific Sets of T- and B-Cell Receptors. J. Allergy Clin. Immunol..

[83] Yaari G., Kleinstein S.H. (2015). Practical Guidelines for B-Cell Receptor Repertoire Sequencing Analysis. Genome Med..

[84] Peng K., Safonova Y., Shugay M., Popejoy A.B., Rodriguez O.L., Breden F., Brodin P., Burkhardt A.M., Bustamante C., Cao-Lormeau V.-M. (2021). Diversity in Immunogenomics: The Value and the Challenge. Nat. Methods.

[85] Rudin C. (2019). Stop Explaining Black Box Machine Learning Models for High Stakes Decisions and Use Interpretable Models Instead. Nat. Mach. Intell..

[86] Vig J., Madani A., Varshney L.R., Xiong C., Socher R., Rajani N.F. BERTology Meets Biology: Interpreting Attention in Protein Language Models. Proceedings of the 9th International Conference on Learning Representations, ICLR 2021.

[87] Meier J., Rao R., Verkuil R., Liu J., Sercu T., Rives A. (2021). Language Models Enable Zero-Shot Prediction of the Effects of Mutations on Protein Function. Proceedings of the 35th International Conference on Neural Information Processing Systems.

[88] Jain S., Wallace B.C. Attention Is Not Explanation. Proceedings of the 2019 Conference of the North American Chapter of the Association for Computational Linguistics: Human Language Technologies, NAACL-HLT 2019.

[89] Springer I., Besser H., Tickotsky-Moskovitz N., Dvorkin S., Louzoun Y. (2020). Prediction of Specific TCR-Peptide Binding From Large Dictionaries of TCR-Peptide Pairs. Front. Immunol..

[90] Saito T., Rehmsmeier M. (2015). The Precision-Recall Plot Is More Informative than the ROC Plot When Evaluating Binary Classifiers on Imbalanced Datasets. PLoS ONE.

[91] Chicco D., Jurman G. (2020). The Advantages of the Matthews Correlation Coefficient (MCC) over F1 Score and Accuracy in Binary Classification Evaluation. BMC Genom..

[92] Greiff V., Weber C.R., Palme J., Bodenhofer U., Miho E., Menzel U., Reddy S.T. (2017). Learning the High-Dimensional Immunogenomic Features That Predict Public and Private Antibody Repertoires. J. Immunol..

[93] Edgar R., Domrachev M., Lash A.E. (2002). Gene Expression Omnibus: NCBI Gene Expression and Hybridization Array Data Repository. Nucleic Acids Res..

[94] Leinonen R., Sugawara H., Shumway M., on behalf of the International Nucleotide Sequence Database Collaboration (2011). The Sequence Read Archive. Nucleic Acids Res..

[95] Leinonen R., Akhtar R., Birney E., Bower L., Cerdeno-Tárraga A., Cheng Y., Cleland I., Faruque N., Goodgame N., Gibson R. (2011). The European Nucleotide Archive. Nucleic Acids Res..

[96] Nolan S., Vignali M., Klinger M., Dines J.N., Kaplan I.M., Svejnoha E., Craft T., Boland K., Pesesky M.W., Gittelman R.M. (2025). A Large-Scale Database of T-Cell Receptor Beta Sequences and Binding Associations from Natural and Synthetic Exposure to SARS-CoV-2. Front. Immunol..

[97] Lefranc M.-P., Giudicelli V., Duroux P., Jabado-Michaloud J., Folch G., Aouinti S., Carillon E., Duvergey H., Houles A., Paysan-Lafosse T. (2015). IMGT^®^, the International ImMunoGeneTics Information System^®^ 25 Years On. Nucleic Acids Res..

[98] Corrie B.D., Marthandan N., Zimonja B., Jaglale J., Zhou Y., Barr E., Knoetze N., Breden F.M.W., Christley S., Scott J.K. (2018). iReceptor: A Platform for Querying and Analyzing Antibody/B-Cell and T-Cell Receptor Repertoire Data across Federated Repositories. Immunol. Rev..

[99] Kovaltsuk A., Leem J., Kelm S., Snowden J., Deane C.M., Krawczyk K. (2018). Observed Antibody Space: A Resource for Data Mining Next-Generation Sequencing of Antibody Repertoires. J. Immunol..

[100] Wu L., Xue Z., Jin S., Zhang J., Guo Y., Bai Y., Jin X., Wang C., Wang L., Liu Z. (2022). huARdb: Human Antigen Receptor Database for Interactive Clonotype-Transcriptome Analysis at the Single-Cell Level. Nucleic Acids Res..

[101] Zhang W., Wang L., Liu K., Wei X., Yang K., Du W., Wang S., Guo N., Ma C., Luo L. (2020). PIRD: Pan Immune Repertoire Database. Bioinformatics.

[102] Rosenfeld A.M., Meng W., Luning Prak E.T., Hershberg U. (2017). ImmuneDB: A System for the Analysis and Exploration of High-Throughput Adaptive Immune Receptor Sequencing Data. Bioinformatics.

[103] Shugay M., Bagaev D.V., Zvyagin I.V., Vroomans R.M., Crawford J.C., Dolton G., Komech E.A., Sycheva A.L., Koneva A.E., Egorov E.S. (2018). VDJdb: A Curated Database of T-Cell Receptor Sequences with Known Antigen Specificity. Nucleic Acids Res..

[104] Vita R., Mahajan S., Overton J.A., Dhanda S.K., Martini S., Cantrell J.R., Wheeler D.K., Sette A., Peters B. (2019). The Immune Epitope Database (IEDB): 2018 Update. Nucleic Acids Res..

[105] Dunbar J., Krawczyk K., Leem J., Baker T., Fuchs A., Georges G., Shi J., Deane C.M. (2014). SAbDab: The Structural Antibody Database. Nucleic Acids Res..

[106] Raybould M.I.J., Kovaltsuk A., Marks C., Deane C.M. (2021). CoV-AbDab: The Coronavirus Antibody Database. Bioinformatics.

[107] Nguyen T., Le H., Quinn T.P., Nguyen T., Le T.D., Venkatesh S. (2021). GraphDTA: Predicting Drug–Target Binding Affinity with Graph Neural Networks. Bioinformatics.

[108] Zhang Z., Chang W.Y., Wang K., Yang Y., Wang X., Yao C., Wu T., Wang L., Wang T. (2022). Interpreting the B-Cell Receptor Repertoire with Single-Cell Gene Expression Using Benisse. Nat. Mach. Intell..

[109] Shan S., Luo S., Yang Z., Hong J., Su Y., Ding F., Fu L., Li C., Chen P., Ma J. (2022). Deep Learning Guided Optimization of Human Antibody against SARS-CoV-2 Variants with Broad Neutralization. Proc. Natl. Acad. Sci. USA.

[110] Yang S., Luo X., Luo J., Jian F., Cao Y. (2025). Predicting SARS-CoV-2 Evolution Dynamics with Spatiotemporal Resolution by DMS-Empowered Protein Language Model. bioRxiv.

[111] Ruffolo J.A., Chu L.-S., Mahajan S.P., Gray J.J. (2023). Fast, Accurate Antibody Structure Prediction from Deep Learning on Massive Set of Natural Antibodies. Nat. Commun..

[112] Yang K.K., Wu Z., Arnold F.H. (2019). Machine-Learning-Guided Directed Evolution for Protein Engineering. Nat. Methods.

[113] Zhou J., Troyanskaya O.G. (2015). Predicting Effects of Noncoding Variants with Deep Learning–Based Sequence Model. Nat. Methods.

[114] Akbar R., Robert P.A., Pavlović M., Jeliazkov J.R., Snapkov I., Slabodkin A., Weber C.R., Scheffer L., Miho E., Haff I.H. (2021). A Compact Vocabulary of Paratope-Epitope Interactions Enables Predictability of Antibody-Antigen Binding. Cell Rep..

[115] Jin R., Ye Q., Wang J., Cao Z., Jiang D., Wang T., Kang Y., Xu W., Hsieh C.-Y., Hou T. (2024). AttABseq: An Attention-Based Deep Learning Prediction Method for Antigen–Antibody Binding Affinity Changes Based on Protein Sequences. Brief. Bioinform..

